# Knowledge and practice of tracheal tube cuff pressure monitoring: a multicenter survey of anaesthesia and critical care providers in a developing country

**DOI:** 10.1186/s13037-021-00311-8

**Published:** 2022-01-14

**Authors:** Arinze Duke George Nwosu, Edmund Ndudi Ossai, Fidelis Anayo Onyekwulu, Adaobi Obianuju Amucheazi, Richard Ewah, Okechukwu Onwuasoigwe, Irene Akhideno

**Affiliations:** 1 Department of Anaesthesia, National Orthopaedic Hospital, Enugu, Nigeria; 2 Department of Community Medicine, Federal Teaching Hospital, Abakaliki, Nigeria; 3 Department of Anaesthesia, University of Nigeria Teaching Hospital, Enugu, Nigeria; 4 Department of Anaesthesia, Federal Teaching Hospital, Abakaliki, Nigeria; 5 Department of Orthopaedic Surgery, University of Nigeria Teaching Hospital, Enugu, Nigeria; 6 Department of Anaesthesia, Irrua Specialist Teaching Hospital, Irrua., Edo state Nigeria

**Keywords:** Anaesthesia, Critical care, Surgery, Tracheal cuff manometer, Nigeria

## Abstract

**Background:**

Tracheal tubes are routinely used during anaesthesia and in the intensive care unit. Subjective monitoring of cuff pressures have been reported to produce consistently inappropriate cuffs pressures, with attendant morbidity. But this practice of unsafe care remains widespread. With the proliferation of intensive care units in Nigeria and increasing access to surgery, morbidity relating to improper tracheal cuff pressure may assume a greater toll. We aimed to evaluate current knowledge and practice of tracheal cuff pressure monitoring among anaesthesia and critical care providers in Nigeria.

**Methods:**

This was a multicenter cross-sectional study conducted from March 18 to April 30, 2021. The first part (A) was conducted at 4 tertiary referral hospitals in Nigeria by means of a self-administered questionnaire on the various cadre of anaesthesia and critical care providers. The second part (B) was a nation-wide telephone survey of anaesthesia faculty fellows affiliated to 13 tertiary hospitals in Nigeria, selected by stratified random sampling.

**Results:**

Only 3.1% (6/196) of the care providers admitted having ever used a tracheal cuff manometer, while 31.1% knew the recommended tracheal cuff pressure. The nationwide telephone survey of anaesthesia faculty fellows revealed that tracheal cuff manometer is neither available, nor has it ever been used in any of the 13 tertiary hospitals surveyed. The ‘Pilot balloon palpation method’ and ‘fixed volume of air from a syringe’ were the most commonly utilized method of cuff pressure estimation by the care providers, at 64.3% and 28.1% respectively in part A survey (84.6% and 15.4% respectively, in the part B survey).

**Conclusion:**

The use of tracheal cuff manometer is very limited among the care providers surveyed in this study. Knowledge regarding tracheal cuff management among the providers is adjudged to be fair, despite the poor practice and unsafe care.

## Background

The tracheal tubes commonly used in anaesthesia, emergency departments and critical care are equipped with cuffs to protect the lower airway from gastric aspiration and other forms of sub-glottic contamination. The cuff also facilitates positive pressure ventilation by forming a good seal with the tracheal mucosa thereby preventing leakage of fresh gas. Over‐inflation of tracheal tube cuff has been associated with postoperative sore throat [1], tracheal oesophageal fistula [2, 3], and tracheal stenosis [4]. Conversely under-inflation of the tracheal cuff has been implicated with ventilator-associated pneumonia (VAP) [5, 6]. The European task force, the American thoracic society and Infectious diseases society of America (ATS-IDSA) guidelines have since recommended cuff pressure (Pcuff) regulation as a measure for controlling ventilator-associated pneumonia in the intensive care unit (ICU) [7].

In the recent, various transducer devices for automated continuous regulation of Pcuff are being introduced owing to concern about periods of suboptimal Pcuff during manual intermittent manometers use, but have not yet shown convincing superiority despite their huge comparative cost [8–10]. The bulkiness and requirement for electricity in these automated continuous regulation devices may have also impacted adversely on their widespread use in clinical practice.

The gold standard for Pcuff monitoring has been the intermittent manual manometer, and guidelines have since recommended that Pcuff should be maintained at 20–30 cm H_2_O [11]. There is preponderance of evidence that tracheal tube cuffs are improperly inflated when manometers are not used [12–14]. Currently, objective measurement of Pcuff is not routinely done in clinical anaesthesia practice, emergency medicine and ICU care in most settings. The unavailability of the manometer which is largely responsible for this shortcoming owes it a lot to the false presumption that the subjective methods of Pcuff monitoring are satisfactory. This applies to the endotracheal tube, but also the tracheostomy tube and laryngeal mask airway. However, with the reports from the United Kingdom and South Africa, it would appear that beside making the manometer available, deficits in knowledge and attitude regarding Pcuff management need to be addressed too [15–17].

With the proliferation of ICUs in Nigeria and increasing access to surgery, morbidity relating to improper tracheal cuff pressure may assume a greater toll. Information regarding the knowledge and practice of Pcuff monitoring by care providers in Nigeria is lacking, but available literature indicate wide variability across the globe. The purpose of our study was to determine the pattern of knowledge and practice of Pcuff management among care providers in Nigeria.

## Methods

### Institutional review board approval

Permission for the survey was granted by the Research ethics committee of National Orthopaedic Hospital, Enugu [IRB: S.313/IV/. Protocol number: 202102003]. The questionnaires were made anonymous in order to conceal the identity of the respondents. Participation was voluntary and the questionnaires were administered to only those who gave verbal informed consent to participate in the survey.

### Study design, setting and participants

This was a multicentre cross-sectional study. The survey was conducted in two parts, between March 18 and April 30, 2021. The first part (A) was facilitated through a structured questionnaire distributed to the care providers (physician anaesthetists, nurse anaesthetists, ICU nurses and anaesthesia technicians) in four tertiary health institutions in Southern Nigeria: National Orthopaedic Hospital, Enugu; University of Nigeria Teaching Hospital, Enugu; Federal Teaching Hospital, Abakaliki; and Irrua Specialist Teaching Hospital, Edo State. The 8-item questionnaire which sought information on basic knowledge and current practice regarding tracheal cuffs among the care providers was pretested and relevant modifications applied. A committee of experts consisting of three anaesthesia faculty fellows deliberated on and allocated the scores to the basic knowledge responses. All the care personnel within the cadre indicated for the study in the four hospitals were eligible, and the total population was to be sampled. The anonymous self-administered questionnaire was distributed among the consenting staff, in–person, by research assistants (placed in sealed envelopes to further enhance anonymity). After recruitment the questionnaires were completed by the respondents, and retrieved immediately. The survey was extended for several days to recruit those who were unavoidably missed on the first day, in order to increase the capture rate. A roll of the eligible personnel in each institution was obtained from the relevant departments in the respective hospitals.

The second part of the study (B) was a nationwide telephone survey. A 3–item structured questionnaire was administered to seek information from anaesthesia faculty fellows regarding the practice of Pcuff management in the respective hospitals, and their individual most applied technique of Pcuff estimation. Using stratified random sampling method, two hospitals per geopolitical zone and one from the federal capital territory, were selected from the institutions that anaesthesia faculty fellows in Nigeria are affiliated to. The four institutions where the care providers were surveyed in the first part of the study were excluded in this second part. Thirteen (13) anaesthesia faculty fellows; one each from the 13 selected health institutions, were contacted by telephone and surveyed.

For both segments of the study all data were recorded on the questionnaire and subsequently captured electronically for ease of analysis.

### Data analysis

The IBM statistical package for social sciences (SPSS) statistical software version 25 was used for data entry and analysis. Categorical variables were summarized using frequencies and proportions. Chi square test of statistical significance was used in the analysis. Difference were considered significant when *p* < 0.05.

Knowledge regarding tracheal cuff management was assessed using responses to the questions in Table 1. In determining good knowledge regarding tracheal cuff pressure management, the variables were assigned different weights based on the recommendation of the Committee of Experts. For example, ‘I consider it necessary to accurately measure tracheal tube cuff pressure’ was scored 20, while knowing the recommended cuff pressure was scored 40, knowing the reason for inflating tracheal tube cuff was scored a total of 20, and being aware that both over-inflation and under-inflation of the cuff could harm the patient was also scored 20. Respondents that scored ≥ 50% of the total score were regarded as having good knowledge regarding tracheal cuff pressure management while those that scored < 50% were classified as having poor knowledge.

**Table 1:** Knowledge regarding tracheal cuff management

Variable	Number of respondents (*n* = 196)	Response rate (%)
**I consider it necessary to accurately measure tracheal tube cuff pressure**		
Yes	173	88.3
No	23	11.7
**Knowledge of recommended tracheal cuff pressure**		
Correct	61	31.1
Incorrect	135	68.9
**The following are harmful to patients**		
Over-inflated cuff only	30	15.3
Under-inflated cuff only	6	3.1
Both over-inflated and under-inflated cuffs	159	81.1
None of the above	1	0.5
**Reason for inflating the tracheal tube cuff**		
** Anchor the tracheal tube**		
Correct	125	63.8
Incorrect	71	36.2
**Protect the airway**		
Correct	158	80.6
Incorrect	38	19.4
**Facilitate ventilation**		
Correct	44	22.4
Incorrect	152	77.6
**Knowledge regarding tracheal cuff management**		
Good	110	56.1
Poor	86	43.9

## Results

The response rate was 88% (38/43) in National Orthopaedic Hospital, Enugu; 76% (61/80) in University of Nigeria Teaching Hospital, Enugu; 73% (55/75) in Federal Teaching Hospital, Abakaliki; and 75% (42/56) in Irrua Specialist Teaching Hospital, Edo State. The overall response rate was 77% (196/254). There was a preponderance of physician anaesthetists (44.9%) in the hospitals surveyed (Table 2).

**Table 2:** Characteristics of the respondents

Variable	Number of respondents (*n* = 196)	Response rate (%)
**Name of Institution**		
University of Nigeria Teaching Hospital Ituku-Ozalla. Enugu	61	31.1
Alex Ekwueme Federal University Teaching Hospital, Abakaliki	55	28.1
Irrua Specialist Teaching Hospital. Edo state	42	21.4
National Orthopedic Hospital, Enugu	38	19.4
**Provider specialty**		
Physician anaesthetist	88	44.9
Nurse anaesthetist	44	22.4
ICU nurse	33	16.8
Anaesthesia technician	31	15.8
**Experience in tracheal tube management (years)**		
˂5 years	78	39.8
5–10 years	69	35.2
> 10 years	49	25.0

### Knowledge regarding tracheal tube cuff management

Only 31.1% of the care providers knew the correct Pcuff to be 20-30 cm H_2_O, while 22.4% were aware that the tracheal tube cuff facilitates mechanical ventilation of intubated patients (Table 1). Most of the care providers were aware that both over-inflated and under-inflated cuffs could harm the patients (Table 1). Fifty-six percent (110/196) of the respondents were adjudged to have good knowledge regarding tracheal tube cuff management (Table 1). The professional cadre of the care provider; *p* < 0.001, but not the number of years of practice; *p* = 0.094, was significantly associated with knowledge regarding tracheal cuff management (Table 3). Among the various professional cadre involved in tracheal cuff care the proportion with good knowledge was highest among the physician anaesthetists, and least among the ICU nurses (Table 3).

**Table 3:** Factors affecting respondent’s knowledge regarding tracheal cuff management

Variable	Knowledge of tracheal cuff pressure (*n* = 196)		χ^2 ^* p* value
	**Good *N* (%)**	**Poor *N* (%)**	
**Experience in tracheal tube management (years)**			
< 5 years	41 (52.6)	37 (47.4)	4.719 0.094
5–10 years	35 (50.7)	34 (49.3)	
> 10 years	34 (69.4)	15 (30.6)	
**Provider specialty**			
Physician anesthetist	62 (70.5)	26 (29.5)	18.772 < 0.001
Nurse anesthetist	18 (40.9)	26 (59.1)	
ICU nurse	11 (33.3)	22 (66.7)	
Anesthesia technician	19 (61.3)	12 (38.7)	

Only 3.1% (6/196) of the care providers have ever used a tracheal cuff manometer. The ‘pilot balloon palpation method’ and ‘fixed volume of air from a syringe’ were the most utilized method of tracheal cuff pressure estimation by most care providers, at 64.3% and 28.1% respectively (Table 4).

**Table 4:** Practice of tracheal cuff management

Variable	Number of respondents (*n* = 196)	Response rate (%)
**Ever used a tracheal tube cuff manometer**		
Yes	6	3.1
No	190	96.9
**Personal method of inflating tracheal cuff**		
Pilot balloon palpation	126	64.3
Fixed volume of air from syringe	55	28.1
Minimal leak technique	2	1.0
Minimal occlusive volume	1	0.5
No method	12	6.1

### Nationwide telephone survey on availability and use of tracheal cuff manometer

The second part of the study (B) was a telephone survey and revealed that tracheal cuff manometer is neither available, nor has it ever been used in any of the 13 tertiary health institutions selected in the national survey. The ‘pilot balloon palpation method’ of cuff pressure estimation was the preferred technique used by 84.6% (11/13) of the anaesthesia faculty fellows interviewed (Table 5).

**Table 5:** National telephone survey on availability and use of tracheal cuff manometer

Variable	Number of respondents (*n* = 13)	Response rate (%)
**Tracheal cuff manometer availability in your center**		
Yes	0	0
No	13	100
**Indicate use of tracheal cuff manometer in your center**		
Regularly	0	0
Sometimes	0	0
Never	13	100
**Personal method of Pcuff estimation**		
Cuff manometer	0	0
Pilot balloon palpation	11	84.6
Fixed volume of air from syringe	2	15.4
Minimal leak technique	0	0
Minimal occlusive volume	0	0
Loss of resistance syringe	0	0
No method	0	0

## Discussion

The study revealed unsafe tracheal cuff management among the surveyed care providers, supported by a weak knowledge base. Previous studies regarding knowledge and practice of trachea cuff pressure management had indicated wide variation across different practice settings around the world, with evidence of substandard practice in most environments. A single-institution questionnare-based study conducted recently in Pakistan had shown that among the critical care and emergency room practitioners of a tertiary hospital 69% of the participants had no prior knowledge about ETT cuff manometer, 73% had never used a manometer while 72% did not know the hazards of inappropriate tracheal cuff pressure [18]. Similarly, among anaesthesia care providers in a military hospital in USA poor knowledge regarding tracheal cuff management was also evident as only 35% of the care providers knew the correct tracheal cuff pressure [19]. In our multicentre study encompassing both anaesthesia and critical care staff only 31.1% of the care providers knew the correct Pcuff, 97% had never used a tracheal cuff manometer, while 81% were aware that improper cuff pressure could harm patients. Meanwhile, a seemingly higher knowledge of the correct tracheal cuff pressure was recorded by 45% of participants in a multicentre questionnaire-based survey conducted among 160 anaesthesiologists practicing in South Africa [15]. In the South African study the participants were all anaesthesiologists (anaesthesia faculty fellows, residents and diplomates), while in our study and the others, the participants were an admixture of physician and non-physician care providers (nurse anaesthetists, certified registered nurse anaesthetists, student registered nurse anaesthetists, anaesthesia technicians, ICU nurses, etc.) who are involved in tracheal cuff management. Hence, the cadre of the participants in the respective surveys may have impacted on the similarities and differences in the knowledge base regarding tracheal cuff management. Thus, 59.6% of respondents had knowledge of the recommended cuff pressure in a study conducted in a Brazilian teaching hospital among consultants and resident anaesthesiologists [20]. It is of note that all the quoted studies were conducted between 2016 and 2021, and could be adjudged to approximate current trends.

The current practice regarding tracheal cuff management in Nigeria is captured in both parts of our study. The questionnaire-based segment (Part1) shows that 97% of the surveyed care providers have never used a tracheal cuff manometer, with the ‘pilot balloon palpation method’ being the most popular method of tracheal cuff pressure estimation. The nationwide telephone survey (Part 2) supports the findings of the questionnaire-based segment regarding the popularity of the ‘pilot balloon palpation method’ of cuff pressure estimation, and the very limited use of tracheal cuff manometer in tracheal cuff management in Nigeria. Whereas all 10 hospitals included in the South African study had tracheal cuff manometers somewhere within their institution, only half of the participants were aware of this and they were not readily available [15]. Their routine technique of tracheal cuff management showed much variability; minimal occlusive volume technique (38.8%); pilot balloon palpation technique (36.3%); minimal leak technique (11.9%); cuff manometer (2.5%). This would suggest that in addition to making the tracheal cuff manometer widely available for airway management, education and change of attitude are necessary in changing the narrative of poor tracheal cuff management. In contrast, among the anaesthesiologists in a Brazilian university teaching hospital 63.8% used the manometer occasionally, while 4.3% used it routinely. All the anesthesiology residents confirmed having used the tracheal cuff manometer, even though it was not regularly available [20]. It is obvious that in spite of the better level of knowledge and practice among the participants in the Brazilian study tracheal cuff pressure management could be said to be currently poor globally. A bi-national survey was conducted in 2019 to evaluate the prevailing practice regarding intraoperative cuff pressure monitoring in private and public hospitals across Australia and New Zealand [21]. Among the 1000 randomly selected anaesthesia faculty fellows, 78.0% submitted that they had ready access to cuff pressure manometer in their hospital, but only 40.0% used them routinely in their practice. Our current national survey of anaesthesia faculty fellows in Nigeria which revealed that the tracheal cuff manometer is neither available, nor has it ever been used in any of the 13 randomly selected tertiary health institutions is thus a far cry.

Several earlier research works had found that most of the healthcare workers who used the palpation technique underestimated the Pcuffs and hyperinflated the tracheal cuffs in both tracheal models and human subjects [20, 22–24]. The palpation technique has remained a very popular technique despite its flaw. It was so unreliable that its use in Iranian ICU patients was associated with universal over-inflation of the tracheal cuff, with all being above 40 cmH_2_O (mean 88.8 ± 27.1 cmH_2_O) [25]. The use of tracheal cuff manometer for objective estimation and monitoring of tracheal cuff pressure reduces postoperative morbidity [1, 26]. The very limited use of the manometer in tracheal cuff management in the country cannot be explained away by mere unavailability since other devices for monitoring patients such as the multi-parameter patient monitors are available. In Nigeria, like in most countries, there are no national or professional guidelines prescribing or mandating the availability or use of objective monitoring of tracheal cuff pressure. The weak knowledge base of the surveyed care providers in Nigeria, as elsewhere, may play a role in sustaining this poor practice. Hence, the need for mass education and enlightenment regarding proper management of the tracheal cuff in intubated patients.

It is of note that the participants in our survey are care providers serving in federal tertiary hospitals, otherwise regarded as elite institutions in the country. Anaesthesia manpower deficit in Nigeria is severe [27], and these same care providers also cater for the bulk of state health institutions and private health facilities. It is therefore considered that their knowledge and practice fairly represent the pattern of tracheal cuff management in Nigeria. The unsafe care regarding tracheal cuff management in Nigeria mirrors the current state of anesthesia safety in some other developing countries [28–30].

A limitation to this study is that it is, like some other cited studies, questionnaire-based, and as such inherently susceptible to response bias, particularly as it pertains to deliberate false responses meant to gratify social desirability. For instance, disclosures regarding practice as volunteered by the participants may not be accurate, or factual. Given that there is unavailability of the tracheal cuff manometer in all the surveyed facilities, the claim by the six respondents to having used the cuff manometer is unsubstantiated. In effect, the proportion of care providers that have ever used the tracheal cuff manometer may be less than the 3% that declared. Furthermore, since the survey took several days to complete in some hospitals, it could have afforded some respondents the opportunity to make reference to texts, or the internet regarding the recommended tracheal cuff pressure and other knowledge questions before being surveyed. Consequently, the actual knowledge base regarding tracheal cuff management may indeed be lower than what we found.

## Conclusion

The use of tracheal cuff manometer is very limited among the surveyed care providers who are involved in tracheal cuff management in Nigeria. Most of the care providers use the subjective “palpation method” which is widely known to give a very poor estimate. Knowledge regarding tracheal cuff management among the providers is adjudged to be fair, despite the poor practice and unsafe care. The knowledge base required to institute and sustain safe tracheal cuff management is currently low. Education could provide awareness and draw attention to this major shortcoming in airway management.

## Data Availability

The datasets analyzed during the current study are available from the corresponding author on reasonable request.

## Abbreviations

**ATS-IDSA**: American thoracic society and Infectious diseases society of America
**ICU**: Intensive care unit
**Pcuff**: Cuff pressure
**SPSS**: Statistical package for social sciences
**VAP**: Ventilator-associated pneumonia
